# Neurocognitive function and health-related quality of life among glioblastoma patients: A prospective study

**DOI:** 10.4102/jphia.v16i1.660

**Published:** 2025-01-10

**Authors:** Mohamed A. Baba, Ahmed Kharbach, Mohamed Lmejjati, Nawal Adali

**Affiliations:** 1 Department of Public Health, Faculty of Medicine and Pharmacy of Agadir, Ibn Zohr University, Agadir, Morocco; 2 Laboratory of Cell Biology and Molecular Genetics, Department of Biology, Faculty of Sciences, Ibn Zohr University, Agadir, Morocco; 3 High Institute of Nursing Professions and Technical Health, Agadir, Morocco; 4 Department of Public Health, Faculty of Medicine and Pharmacy of Rabat, Mohammed V University, Rabat, Morocco; 5 Department of Neurosurgery, Faculty of Medicine and Pharmacy of Agadir, Ibn Zohr University, Agadir, Morocco; 6 Department of Neurology, Faculty of Medicine and Pharmacy of Agadir, Ibn Zohr University, Agadir, Morocco

**Keywords:** cancer, glioblastoma, brain, quality of life, Morocco

## Abstract

**Background:**

Glioblastomas are aggressive brain tumours that significantly impact patients’ functional and psychological well-being, making the evaluation of health-related quality of life (HRQoL) essential.

**Aim:**

This study aims to assess the quality of life (QoL) and neurocognitive status of glioblastoma patients in Morocco.

**Setting:**

Patients were recruited from two renowned oncology centres in Morocco.

**Methods:**

Patients receiving care at two renowned oncology centres were enrolled between April 2021 and January 2024. The QLQ-C30 and QLQ-BN20 scales were used to assess QoL, while neurocognitive function was evaluated using the Mini-Mental State Examination (MMSE) test. Data normality were checked using the Shapiro-Wilk test, and non-parametric methods were used to compare scores. Variables such as age, tumour laterality, residence, and educational status were examined for their association with overall QoL. Both univariate linear regression and multivariate regression analyses were conducted. Statistical analyses were performed using the JAMOVI software.

**Results:**

The study enrolled 106 patients, with a median age of 56.7 years. Among them, 66% were male, while 34% were female. Multiple linear regression showed that age (B: –15.46; 95% CI: –24.15 to –6.77; *p* < 0.001), education level (B: –9.36; 95% CI: 0.84 to 17.88; *p* = 0.032), distance from hospital (B: –9.85; 95% CI: –18.34 to –1.36; *p* = 0.023), and tumour laterality (B= –12.36; 95% CI: –20.65 to –4.06; *p* = 0.004) were significantly associated with overall QoL at 3 months follow-up.

**Conclusion:**

The results indicate a decline in HRQoL during follow-up among glioblastoma patients. Advancing age, tumour location on the left side, education level, and proximity to the hospital significantly influence the overall QoL.

**Contribution:**

This study highlights the critical factors impacting HRQoL in glioblastoma patients in Morocco, providing insights for improving patient care.

## Introduction

Gliomas account for more than 80% of all brain tumours.^1^ According to the World Health Organization (WHO), they are classified based on their cell of origin.^2^ The glioblastoma multiforme (GBM) is one of the most malignant brain tumours in humans, accounting for approximately 60% of all brain tumours in adults.^1^ The incidence varies according to epidemiological reports, ranging from 3.19 cases per 100 000 person-years^3,4,5^ to 4.17 cases per 100 000 person-years.^6,7,8,9^ In Morocco, there is no national cancer registry, and the regional registries (Casablanca and Rabat) do not have specific epidemiological descriptive data on glioblastoma.^10^ This absence of systematic surveillance impedes a comprehensive evaluation of the prevalence, incidence and clinical outcomes of patients affected by this condition, thereby creating a substantial gap in the medical literature and clinical practice.

The GBM is also associated with a poor prognosis. Despite aggressive surgery, radiation and chemotherapies with temozolomide, the average survival time remains approximately one year.^10^ Even with advancements in therapeutic approaches, the importance of quality of life (QoL) in the management of glioblastoma patients has become increasingly significant. This is because of the grim prognosis associated with this condition, compounded by the concurrent effects of both the tumour and its treatment on the individual’s functional, psychological and social well-being. Hence, there’s a critical need to incorporate the concept of health-related quality of life (HRQoL) into the management of patients.^11,12,13^

The treatment of glioblastoma in Morocco presents significant challenges. Access to multidisciplinary care and advanced therapies, such as concomitant chemotherapy and radiosurgery treatments, remains limited in many regions of the country. Furthermore, healthcare infrastructure is often unevenly distributed, complicating timely access to treatment for many patients, particularly those residing in rural areas far from major medical centres. This context directly impacts the prognosis and QoL of Moroccan patients.

Health-related quality of life encompasses several dimensions related to the physical, social and emotional health of the individual and has become an important element of medical decision-making along with the efficacy and safety of treatments.^14^ It has been found that HRQoL assessments conducted by patients differ from that seen by treating physicians and therefore the patient has the ability to assess more accurately their QoL at a given moment of the disease.^15^

The majority of studies on the QoL of patients with glioblastoma come from countries with highly developed healthcare systems and advanced medical infrastructure, which limits the generalisability of the findings to other contexts, particularly that of Morocco. Furthermore, a recent systematic review focussing on Mediterranean countries revealed a lack of published studies evaluating the HRQoL of patients with glioblastoma in the Maghreb region, including Morocco.^16^ In this perspective, the study aimed to assess the QoL and neurocognitive state in Moroccan patients with glioblastoma.

## Research methods and design

### Study design

This study is a longitudinal, observational study aimed at assessing the QoL and neurocognitive status in patients with glioblastoma.

### Patient selection

Individuals newly diagnosed with glioblastoma were recruited from two oncology reference centres (public and private) situated in the southern region of Morocco. Participants were recruited between April 2021 and January 2024, and included participants over 18 years of age and fluent in dialectal Arabic.

All patients included in our study were confirmed to have glioblastoma through both biopsy and imaging. Each patient underwent surgical intervention and was scheduled for either adjuvant radiotherapy alone or concurrent chemotherapy and radiotherapy (CCR)

### Collection of data

The participants were required to fill out the questionnaires at three specific points in time, namely at baseline: prior to the commencement of radiotherapy, on completion of radiotherapy and again three months post-radiotherapy. The scores recorded at each of these instances were incorporated into the statistical analysis. T0: initial evaluation, T1: end of radiotherapy, T2: 3 months after radiotherapy.

### Demographic characteristics and Karnofsky Performance Status evaluation

Demographic characteristics of the patients, including age, gender, marital status, place of residence (urban or rural) and level of education, were collected using a structured survey form. In addition to these demographic variables, the Karnofsky Performance Status (KPS) was assessed at baseline to evaluate the functional status of each patient. The KPS score, ranging from 0 to 100, was recorded to measure the patients’ ability to carry out daily activities, with lower scores indicating greater functional impairment.^17^

### Assessment of health-related quality of life

Two questionnaires, the European Organization for Research and Treatment of Cancer Quality of Life Questionnaire (EORT CQLQC30) version 3.0 and the EORTC QLQ-Brain Cancer Module (QLQ-BN20) were selected to assess the QoL.

For the evaluation of the neurocognitive status of the patients, the Mini-Mental State Examination test (MMSE) was used.

The EORTC QLQ-C30 version 3.0 is a validated questionnaire used to assesses the overall health-related QoL among individuals diagnosed with cancer. The questionnaire is divided into 30 items to evaluate five functional dimensions, three symptom scales and provide an overall health scale/ Health-Related Quality of Life (HRQoL).^18^ The scale has already been translated and validated in Moroccan Arabic dialect.^19^

The EORTC QLQ-BN20 includes 20 items assessing communication deficit, motor dysfunction, visual disorder, other various disease symptoms, treatment toxicities and future uncertainty. European Organization for Research and Treatment of Cancer C30 and BN-20 questionnaires items were scaled and scored using recommended EORTC procedures.^18,20^ The QLQ-BN20 has already been translated and validated in the Moroccan Arabic dialect.^21^

Mini-Mental State Examination or Folstein test,^22^ is a brief 30-item questionnaire to screen for cognitive impairment and at the same time to follow changes in these impairments over time. It is a scale already translated and validated in the Moroccan Arabic dialect.^23^

### Clarification of variables

In this study, we identified and analysed a range of variables to understand their impact on the global health status score. The explanatory (independent) variables considered were: age, gender, educational level, marital status, distance from hospital, KPS, MMSE and tumour laterality. These variables were selected based on their potential influence on the patients’ overall health outcomes. The dependent (outcome) variable was the global health status score, a component of the EORTC-C30, which provides a comprehensive measure of QoL. By examining these relationships, we aimed to identify key factors that affect the global health status of patients with glioblastoma.

### Statistical analysis

Questionnaire scores were expressed as mean (± standard deviation [s.d.]) and median (interquartile range [IQR]). Descriptive results of QoL scores were expressed as mean (s.d.). The scores had a non-normal distribution and were therefore compared by non-parametric methods. Data normality were checked using the Shapiro–Wilk test.

Friedman’s test was employed for whole group comparisons, evaluating numerical differences between consecutive measurements in dependent groups. For pairwise comparisons within dependent groups, the Wilcoxon test was utilised. Additionally, quantitative variables with consecutive measurements underwent assessment either via the Friedman test or the Wilcoxon test. For numerical comparisons among independent groups, the Mann–Whitney U test or Kruskal–Wallis test were applied, depending on whether the groups were paired or unpaired.

Univariate and multivariate analyses were conducted to determine the factors influencing the overall QoL score at 3 months of follow-up. All independent variables with a *p*-value < 0.20 in the univariate analysis were included in the subsequent multivariate linear regression analysis. *p*-values < 0.05 were considered indicative of statistical significance.

Statistical analysis was performed using JAMOVI 2.3.26 (Jamovi Project, Sydney, New South Wales, Australia).

### Ethical considerations

Ethical approval to conduct this study was obtained from the Faculty of Medicine and Pharmacy of Agadir Ethics Committee (No. 01/21).

All participants gave their informed consent for inclusion before they participated in the study. The study was conducted in accordance with the Declaration of Helsinki.

## Results

A total of 112 patients were enrolled; however, 6 withdrew during the evaluation process, resulting in a final inclusion of 106 patients in the study. Table 1 presents the fundamental demographic characteristics. The median age was 56.7 years. Of these, 66.0% were male, while 34.0% were female. The Karnofsky Performance Index was ≥ 80 in 59 patients and < 80 in 47 patients. Most patients were in a relationship, accounting for 69.8% of the total sample (Table 1).

**TABLE 1:** Distribution by demographic characteristics, residence, tumour laterality, baseline mini-mental state examination and Karnofsky scores.

Variables	Frequency (*n*)	%	Mean ± s.d.
**Age (years)**	-	-	56.7 ± 11.5
< 55	49	46.2	-
≥ 55	57	53.8	-
**Gender**			
Male	70	66.0	-
Female	36	34.0	-
**Marital status**			
Without partner	32	30.2	-
With partner	74	69.8	-
**Localisation of the tumour**			
Left	59	55.7	-
Right	47	44.3	-
**The location of the treatment centre**			
In the city of residence	48	45.3	-
Outside the city of residence	58	54.7	-
**KPS†**			
< 80	47	44.3	-
≥ 80	59	55.7	-
**MMSE**			
≤ 27	33	31.1	-
>27	73	68.9	-

MMSE, mini-mental state examination; KPS, Karnofsky performance status.

† , The baseline measures.

**FIGURE 1:**
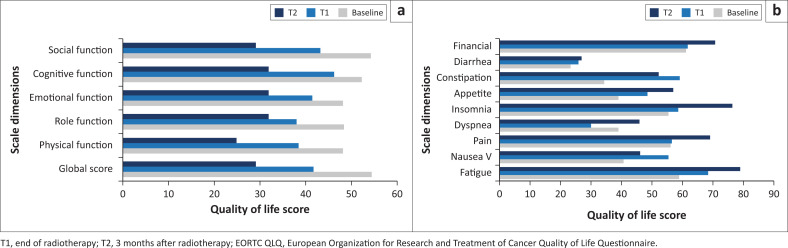
Baseline and follow-up EORTC-C30 scores of the study population (*n* = 106). (a) Baseline and follow-up global score and functional scales (physical, function role, emotional function, cognitive function and social function) of EORTC QLQ-C30. (b) The EORTC QLQ-C30 baseline and follow-up symptom scales (pain, dyspnoea, insomnia, loss of appetite, constipation, diarrhoea, and financial impact).

Baseline and follow-up scores for the EORTC QLQ-C30 are shown in Figure 1 (a and b). During follow-up, the mean scores for the global and functional domains of the EORTC QLQ-C30 decreased, especially for the physical, cognitive and global functioning scores, while the Symptom Domain scores increased with a notable increase in the fatigue, insomnia and funding scores (Figure 1).

In addition to the findings from the QLQ-C30, the scores from the BN-20 also exhibited a corresponding increase. BN-20 symptom scores significantly increased during follow-up compared to baseline, especially for somnolence, visual disorder and motor dysfunction scores. The comparison of the scores between the different measurement times revealed a difference that was statistically significant *p* < 0.05 for almost all the symptoms except for communication deficit and seizure scores (Table 2).

**TABLE 2:** Quality of life score of studied patients during follow-up using BN20 (*n* = 106).

Dimension	Baseline (Mean ± s.d.)	T0 (Mean ± s.d.)	T1 (Mean ± s.d.)	*p* *
Future U	45.23 ± 31.02	31.84 ± 27.69	48.53 ± 35.49	**0.012**
Visual D	38.80 ± 25.07	68.25 ± 25.70	54.67 ± 34.06	**0.002**
Motor D	50.61 ± 19.93	75.66 ± 25.54	61.37 ± 27.13	**0.001**
Communication D	48.50 ± 19.78	54.65 ± 30.21	52.20 ± 22.27	0.706
Head A	75.13 ± 25.37	50.22 ± 23.85	74.07 ± 25.01	**0.001**
Seizures	47.08 ± 19.52	52.67 ± 19.79	54.97 ± 23.28	0.218
Drowsiness	50.10 ± 22.21	55.98 ± 23.77	64.55 ± 29.55	**0.003**
Hair l	24.29 ± 22.54	65.57 ± 27.63	58.20 ± 26.75	**0.001**
Itchy s	47.69 ± 17.67	59.04 ± 25.13	54.49 ± 24.17	**0.013**
Leg w	47.08 ± 18.58	55.01 ± 25.50	60.81 ± 27.77	**0.009**
Bladder C	39.68 ± 24.58	45.50 ± 27.63	47.77 ± 29.15	**0.020**

Note: Data in bold is statistically significant.

T1, end of radiotherapy; T2, 3 months after radiotherapy; s.d., standard deviation; IQR, interquartile range.

* , Friedman’s test.

The scores for future uncertainty significantly increased, indicating that patients felt more uncertain about their future as the follow-up progressed (*p* = 0.012). Similarly, the visual disorder scores significantly increased during follow-up, indicating a deterioration in patients’ vision (*p* = 0.002). In addition, there was a significant increase in motor dysfunction scores, suggesting a worsening of patients’ motor abilities (*p* = 0.001). Furthermore, the drowsiness scores significantly increased, reflecting an increase in fatigue and sleepiness among patients (*p* = 0.003). The headache scores also showed a significant increase, indicating a worsening of headaches among patients (*p* = 0.001). The leg weakness scores significantly increased, suggesting a deterioration in patients’ leg strength (*p* = 0.009). Moreover, the hair loss scores significantly increased, indicating a worsening of hair loss among patients (*p* = 0.001). The itchy skin scores significantly increased, indicating a worsening of skin itchiness among patients (*p* = 0.013). Lastly, the bladder control scores showed a significant increase, indicating a worsening of bladder control among patients (*p* = 0.020).

On the other hand, the communication deficit scores and the seizure scores did not show a significant change, indicating stability in this dimension (*p* = 0.706 and *p* = 0.218, respectively) (Table 2).

The statistical analysis revealed a significant difference between the overall QoL score and the individual patient characteristics, particularly in relation to age (43.50 ± 19.50 vs 22.80 ± 24.50, *p* < 0.001), location of the tumour (37.30 ± 20.60 vs. 26.20 ± 27.70, *p* = 0.025) and location of the treatment centre (38.90 ± 24.20 vs 27.0 ± 23.70, *p* = 0.012). However, gender and marital status did not reveal a statistically significant difference (Table 3).

**TABLE 3:** Comparison of global quality of life scores with patient characteristics (*n* = 106).

Variables	Global health status score	
	Mean ± s.d.	*p*
**Age (years)**	-	< 0.001*
< 55	43.5 ± 19.5	-
≥ 55	22.8 ± 24.5	-
**Gender**	-	0.466*
Male	33.8 ± 22.3	-
Female	30.0 ± 28.0	-
**Marital status**	-	0.378*
Without partner	29.20 ± 22.00	-
With partner	33.80 ± 25.60	-
**Tumour location**	-	0.025*
Left	37.30 ± 20.60	-
Right	26.20 ± 27.70	-
**The location of the treatment centre**	-	0.012**
In the city of residence	38.9 ± 24.20	-
Outside the city of residence	27.0 ± 23.70	-

Note: Data in bold is statistically significant.

* , Student’s test;

** , Welsh test.

The global function scores from the QLQ-C30 and the neurocognitive status assessment scores both declined over time. Notably, a significant positive correlation was observed between the global QoL scores and neurocognitive function scores at each follow-up (*r* = 0.561, *p* < 0.001 at T1; *r* = 0.313, *p* < 0.001 at T2) (Table 4).

**TABLE 4:** Correlation analysis of global scores (EORTC C30) and cognitive function (Mini-Mental State Examination) (*N* = 106).

Neurocognitive status score (MMSE)	Global health status score		
	Baseline T0	T1	T2
**Baseline T0**			
*r*	0.489	-	-
*p*	< 0.001*	-	-
**T1**			
*r*	-	0.561	-
*p*	-	< 0.001*	-
**T2**			
*r*	-	-	0.313
*p*	-	-	0.001*

T0, initial evaluation; T1, end of radiotherapy; T2, 3 months after radiotherapy; MMSE, mini-mental state examination.

* , Spearman’s correlation analysis.

### Factors influencing the overall quality of life of patients

Univariate analysis first revealed that: age (B: –20.4; 95% confidence interval [CI]: –29.4 to –12.1; *p* < 0.001), level of education (B: 14.9; 95% CI: 5.75 to 24.0; *p* = 0.002], distance from hospital (B: –11.9; 95% CI: –21.1 to –2.61; *p* = 0.012), tumour laterality (B: –11.1; 95% CI: –20.4 to –1.75; *p* = 0.020) were significantly associated with the overall QoL score.

These significant variables were subsequently introduced into the multivariate model. The results showed that the following factors were significantly associated with the overall QoL score at 3 months of follow-up: age (B: –15.46; 95% CI: –24.15 to –6.77; *p* < 0.001), level of education (B: 9.36; 95% CI: 0.84 to 17.88; *p* = 0.032], distance from the hospital [B: –9.85; 95% CI: –18.34 to –1.36; *p* = 0.023], and the laterality of the tumour (B= –12.36; 95% CI: –20.65 to –4.06; *p* = 0.004) (Table 5).

**TABLE 5:** Univariate and multivariate analysis of factors influencing global quality of life (EORTC QLQ-C30) (*N* = 106).

Variable	Comparison	Global health status score					
		Univariate†			Multivariate†		
		*β*	95% CI	*p*	*β*	95% CI	*p*
Age	≥ 55 to < 55	**−20.700**	**−29.4 to −12.1**	**< 0.001**	−15.46	−24.15 to −6.77	**< 0.001**
Gender	M – F	3.820	−5.95 to 13.6	0.440	-	-	-
Educational level	Illiterate – With education	**−14.900**	**−24.0 to −5.75**	**0.002**	−9.36	−17.9 to −0.842	**0.032**
Marital status	With_ Without	4.600	−5.70-14.9	0.378		-	-
Distance from Hospital	Out of town – in town	**−11.900**	**−21.1 to −2.61**	**0.012**	−9.85	−18.34 to −1.36	**0.023**
KPS	> 80 to ≤ 80	−0.204	−0.715 to 0.306	0.427	-	-	-
MMSE	> 27 to ≤ 27	1.550	−8.70 to 11.8	0.765	-	-	-
Laterality of the tumour	R–L	**−11.100**	**−20.4 to −1.75**	**0.020**	−12.36	−20.65 to −4.06	**0.004**

Note: Data in bold is statistically significant.

*β*, beta; CI, confidence interval; M, male; F, female; R, Right; L, Left; MMSE, mini-mental state examination; KPS, Karnofsky Performance Status.

† , Linear regression.

This suggests that, in this study, factors such as being 55 years of age or older, residing outside the city of the treatment centre, having no education and having a tumour located on the right side are all potentially associated with a greater reduction in the QoL among Moroccan patients with glioblastomas in the current series.

## Discussion

The main aim of this study is to assess the HRQoL and neurocognitive function in Moroccan patients diagnosed with glioblastoma. This research holds significant importance as it pioneers the examination of QoL specifically within the Moroccan population, thereby addressing a gap identified in a recent systematic review that pointed out the lack of studies assessing the QoL of individuals with glioblastoma in Morocco.^16^ Furthermore, considering the scarcity of data regarding the HRQoL among patients with gliomas in the literature, it’s noteworthy that several studies have demonstrated variations in HRQoL among patient populations stemming from diverse geographical, social and cultural backgrounds.^24,25^

The baseline EORTC C-30 and BN-20 scores of Moroccan patients with glioblastoma were lower than those of patients living in Europe,^26^ but were almost similar to those of patients living in India in Turkey.^24,27^

In this study, statistical significance was observed in the average global QoL score concerning different individual patient characteristics. Particularly, regarding age, older patients displayed lower scores compared to their younger counterparts. This finding aligns with numerous previous studies that have consistently highlighted the adverse effect on the QoL on among glioblastoma patients.^28,29,30^ The tumour’s left-sided localisation has been associated with a detrimental impact on QoL. Nevertheless, existing literature presents conflicting findings. Some studies have supported the notion that left lateralisation negatively affects various dimensions of HRQoL.^31,32,33^ Additional research has suggested that tumours on the right side have a greater impact on QoL,^34^ Conversely, a separate study found that tumour lateralisation did not have any effect on any dimension of QoL.^35^

The findings of this study demonstrated decreased overall QoL scores among patients residing at a considerable distance from the treatment hospital; however, the association between distance from the treatment hospital and patients’ QoL has been poorly documented in the literature. A recent Australian study highlighted a negative correlation between remoteness and cancer-specific QoL.^36^ Moreover, an additional investigation conducted in Ireland revealed that dimensions of QoL among patients with colorectal cancer are diminished among survivors residing at a distance from the treatment hospital.^37^

Certainly, this negative effect of remoteness on QoL can be explained by the burden of daily travel to treatment centres and taking into account the cost and time of transport, in very specific patients with glioblastoma who present several handicaps and disabilities during the disease.

Regarding the neurocognitive status of patients, the findings indicated that both the QoL and neurocognitive state underwent changes throughout the disease follow-up period among those with glioblastoma. Moreover, the results demonstrated a notable correlation between these factors. In line with these findings, several prior studies have suggested that cognitive decline serves as a primary indicator of unfavourable disease progression, potentially impacting QoL as well.^16,38^

In practice, the results of this study emphasise the importance of integrating routine HRQoL assessments into the treatment protocols for glioblastoma patients in the region. This would allow healthcare providers to track changes in both the neurocognitive and overall well-being of their patients, thus facilitating timely supportive interventions. Moreover, the identification of factors such as age, tumour laterality and education level as determinants of QoL could guide personalised patient management strategies.

Future research should focus on longitudinal studies that explore the long-term effects of glioblastoma treatment on patients’ cognitive and functional outcomes. In addition, there is a need to investigate potential interventions aimed at mitigating neurocognitive decline and improving HRQoL. Expanding this research to include a larger and more diverse patient population across different regions of Morocco and North Africa would further enhance the generalisability of the findings.

### Limitations of the study

Despite the valuable information gained from this study, several limitations must be acknowledged. These include its regional focus, which may limit the generalisability of the results to other populations. Additionally, there is a potential bias because of the participation of patients with low MMSE scores, which could have affected their ability to understand and respond accurately to the questionnaires, thereby influencing the results. Furthermore, a longer longitudinal follow-up would be necessary to thoroughly assess the long-term impact of treatment on the QoL and neurocognitive status of patients, especially given the aggressive nature of glioblastoma.

## Conclusion

In conclusion, to our knowledge, this study is the first to report on HRQoL in patients with glioblastoma within a Moroccan population. Our results reveal a deterioration in HRQoL among patients with glioblastoma as time progresses. Various factors including older age, left-sided tumour location, level of education and distance from the hospital, have been identified as determinants influencing the overall QoL of patients. Moreover, a significant correlation between the overall QoL score and the neurocognitive state of patients during the disease course was observed.

Given these findings, it is crucial to incorporate regular assessments of both HRQoL and neurocognitive function into the routine care of glioblastoma patients. Healthcare providers should prioritise interventions that address the specific needs of patients based on age, tumour location and socio-economic factors such as access to care. Policymakers should consider strategies to improve access to oncology services, especially in remote areas, and invest in educational programmes to support patients and their families in managing the psychological and cognitive challenges associated with the disease.

Future studies should focus on larger, multicentre cohorts to enhance the generalisability of the findings. A longer follow-up period would also be valuable to better understand the long-term impact of treatments on HRQoL and neurocognitive status. Furthermore, exploring targeted interventions to mitigate the decline in HRQoL, particularly in older patients and those with left-sided tumours, could provide new insights into improving patient outcomes.
